# Adaptation and validation of a culturally adapted HIV stigma scale in Myanmar

**DOI:** 10.1186/s12889-021-11685-w

**Published:** 2021-09-13

**Authors:** Feifei Huang, Wei-Ti Chen, Cheng-Shi Shiu, Sai Htun Lin, Min San Tun, Thet Wai Nwe, Yin Thet Nu Oo, Htun Nyunt Oo

**Affiliations:** 1 School of Nursing, Fujian Medical University, Fuzhou, China; 2 School of Nursing, University of California Los Angeles, Los Angeles, CA 90095 USA; 3 Department of Social Work, National Taiwan University, Taipei, Taiwan; 4 Advocacy, Human Right & Technical Services Department, Secretariat Office Myanmar Positive Group (MPG), Yangon, Myanmar; 5 Department of Public Health, Ministry of Health and Sports, National AIDS Program, Naypyitaw, 15011 Myanmar; 6 Department of Medical Research, Deputy Director, Health System Research Division, Yangon, Myanmar

**Keywords:** HIV, Stigma, Myanmar, Psychometrics, Rasch analysis

## Abstract

**Background:**

HIV stigma is a common barrier to HIV prevention, testing, and treatment adherence, especially for low- and middle-income countries such as Myanmar. However, there was no validated Myanmar version of a stigma scale.

Therefore, we adapted the English version of the 40-item Berger’s HIV stigma scale and the 7-item Indian HIV stigma scale into a 47-item Myanmar HIV stigma scale and then evaluated the scale’s psychometric properties.

**Method:**

From January 2020 to May 2020, using random sampling methods, 216 eligible Myanmar people living with HIV/AIDS (PLWHA) were contacted from a closed Facebook group that included more than 10,000 PLWHA. A sample of 156 Myanmar PLWHA completed the online self-reported survey.

**Results:**

A six-factor structure for the scale was determined through exploratory factor analysis, explaining 68.23% of the total variance. After deleting 12 items, the 35-item HIV stigma scale achieved Cronbach ‘s α of 0.72 to 0.95. Construct validity of the scale was demonstrated by significant association with self-reported depression and social support levels (r = 0.60, and − 0.77, *p* < 0.01). In Rasch analysis, the scale achieved person reliability of 3.40 and 1.53 and a separation index of 0.92 and 0.70. The infit and outfit mean squares for each item ranged from 0.68 to 1.40. No differential item functioning across gender or educational level was found.

**Conclusions:**

The psychometric properties of the 35-item Myanmar version of the HIV stigma scale support it as a measure of stigma among PLWHA in Myanmar. This instrument could help healthcare providers to better understand how stigma operates in PLWHA and to develop tailored stigma-reduction interventions in Myanmar.

**Supplementary Information:**

The online version contains supplementary material available at 10.1186/s12889-021-11685-w.

## Background

HIV has become one of the major public health challenges contributing to high disease burden globally, especially for low- and middle-income countries such as Myanmar [1]. After Thailand, Myanmar has the second-highest HIV prevalence in Southeast Asia: 0.8%. In 2018, there were an estimated 240,000 people living with HIV/AIDS (PLWHA) in Myanmar, with 11,000 new infections and 7800 deaths [2]. More than 70% of new HIV infections in Myanmar were among people who inject drugs (PWID), men who have sex with men (MSM), and sex workers who were transgender, all of whom mostly live in urban areas (e.g., Mandalay, Yangon, Sagaing, Kachin, and Shan North) [3]. Due to the alarming HIV epidemic in Myanmar, UNAIDS classified it as a “fast-track” country in 2014 to rapidly scale-up the HIV prevention, testing, and treatment programs, although progress in these areas has been uneven [4]. As a resource-limited country, only 70% of PLWHA in Myanmar were in treatment in 2020 [2], which falls short of the UNAIDS “90–90-90” target goals [5].

HIV stigma—which refers to prejudice against, discrimination toward, and discrediting PLWHA and the groups or communities with which PLWHA associate—is a common barrier to HIV prevention, testing, and treatment adherence [6, 7]. HIV stigma is considered to be a fundamental cause of health inequalities and poor health outcomes [1, 8]. Studies have shown that stigma is associated with non-disclosure [9], delayed healthcare-seeking [9, 10], lower treatment adherence [11], faster disease progression, and poorer mental health (e.g., depression, lower satisfaction with life) [12, 13]. Subsequently, such stigma contributes to lower quality of life (QOL) [1].

Currently, there are limited social support systems in Myanmar for PLWHA. As a result of their serostatus, many PLWHA encounter stigma and discrimination from their family, community, and healthcare systems [1, 12, 14]. One study evaluating hospital accessibility in Myanmar found that PLWHA were relegated to segregated waiting areas and wards after their HIV serostatus was discovered [15]. To end the public health threat of HIV and provide services that safeguard and encourage human rights for all, Myanmar developed the National Strategic Plan on HIV and AIDS (2016–2020) (National Strategic Plan, NSP III), which focuses on five strategic milestones, including that “90% of people living with, at risk of and affected by HIV report no discrimination, especially in health, education and workplace settings [16].”

Most of the stigma scales used in recent studies in Myanmar were widely used in Western countries [17, 18], and only one scale, which we developed, was adapted from an Indian HIV stigma scale [19]. However, efforts to reduce HIV-related stigma have not yet matched the magnitude of the problem [20]. The clear lack of evidence on how to reduce the stigma of PLWHA in Myanmar might be because of the lack of valid tools to evaluate HIV stigma [1].

HIV stigma can be enacted, anticipated, or internalized, and it is important that an instrument can identify and differentiate these stigma mechanisms. With such information, researchers could design a culturally sensitive intervention to decrease HIV stigma [21]. Several instruments are currently used to measure HIV-related stigma, especially the 40-item HIV stigma scale developed by Berger et al. (2001), one of the commonly used instruments that cover the three stigma mechanisms affecting PLWHA [22]. The Berger’ s HIV stigma scale had been translated into several language versions, such as Spanish, Swedish, Chinese, and South India [9, 23–26]—but not yet in the Myanmar Burmese language.

Stigma is related to a specific context of culture and power [7, 20]. Take disclosure concerns for example, compared to Swedish PLWHA [27], Indian families are far more involved in the care of their members in the Indian society; this makes it more difficult to keep the HIV serostatus information within the family [20]. The stigma that may result from this is influenced by cultural differences. Comparing India and Sweden, India has been characterized as a collectivist society, and Sweden more of an individualistic society, particularly regarding interpersonal issues [27]. This may lead to the difference stigma experience of PLHIV. Thus, the HIV stigma scale, which was developed in the United States needs to be adapted and tested to ensure its sensitivities for use in other cultural contexts [1]. In other words, HIV-related stigma is culturally specific and influenced by local cultural beliefs [20].

Myanmar, which lies on the Southeast Asian mainland bordered by Bangladesh, China, India, Laos, and Thailand, has a rich history influenced by British expansionism in the 19th and early 20th centuries [28]. Ethnically, Myanmar is a multi-religious country that includes communities of Muslims, Hindus, Buddhists, and Catholics [1]. These local religious ideologies provide the framework around which individuals and society interpret and address their suffering from chronic illnesses such as HIV and mental disorders [28]. For example, for Buddhists, illness is often explained in terms of karma, or cause and effect [29]. Therefore, in addition to Berger’s HIV stigma scale, we also adapted items focusing on religious and vicarious stigma (i.e., stories of discrimination experienced by others) from an HIV stigma scale tested in India to complete a culturally appropriate instrument that measures HIV stigma among PLWHA in Myanmar [27].

To understand the mechanisms and status quo of HIV stigma experienced by PLWHA in Myanmar, the current study’s aim was to (1) describe a culturally appropriate scale to measure their HIV stigma, adapted from Berger’s HIV stigma scale [22] and some of the items from the Indian HIV stigma scale [27], and (2) evaluate the psychometric properties of the scale with both Classical Test Theory (CTT) and Rasch Analysis.

## Methods

### Design

This cross-sectional descriptive study was approved by the relevant institutional review boards and was conducted in Myanmar from January 2020 to May 2020. We culturally adapted the Berger and Indian HIV stigma scales to create the Myanmar version of the HIV stigma scale and examined the psychometric properties of the scale, which were adherent to COSMIN (**CO**nsensus-based **S**tandards for the selection of health status **M**easurement **In**struments) checklist [30]. The cross-sectional survey adhered to the Strengthening the Reporting of Observational Studies in Epidemiology (STROBE) statement [31].

### Participants

A sample of 216 eligible PLWHA was recruited from a closed Facebook group that included more than 10,000 Myanmar residents, more than 90% of whom were PLWHA. The remaining members were family members of PLWHA or HIV-related workers who answered members’ questions. All participants lived in Myanmar and were at least 18 years of age, were diagnosed with HIV, were able to provide informed consent, and could read, write, and use the internet online survey instrument.

### Sampling

The administrators of the Facebook group were healthcare providers and HIV peer group volunteers. By using random sampling methods, the researcher contacted one PLWHA for every five individuals on the site of the Facebook roster until the targeted sample size was achieved. If participants agreed to participate and were able to provide informed consent, an individualized survey link was sent to them via the institutional Research Electronic Data Capture (REDCap) system.

### Developing the Myanmar version of the HIV stigma scale

We adapted the two HIV stigma scales to measure the stigma experienced by PLWHA in Myanmar in the following four stages:

#### Phase 1

##### Item exploration

To create the 47-item HIV stigma scale in Myanmar (HIVSS-M-1), we adapted the 40-item Berger’s HIV stigma scale [22] (a sample item being, “I worry people who know I have HIV will tell others”) and 7 items from the Indian HIV stigma scale [27] (two sample items being, “I feel that I am paying for karma or sins because you have HIV.” “I’ve been refused medical care or denied hospital services because I have HIV.”). All of the items were rated using a four-point Likert scale (1 = “strongly disagree,” 2 = “disagree,” 3 = “agree,” 4 = “strongly agree”).

#### Phase 2

##### Translation

We adapted Brislin’s translation model for cross-cultural translation, which comprises translation, back-translation, comparison, and linguistic adaption [32, 33]. The HIVSS-M-1 was translated independently from English into Myanmar by a bilingual physician who was providing HIV care in Myanmar. Then, a bilingual researcher (Myanmar-English) back-translated the Myanmar version into English. Both of the translators were native Burmese who studied and worked in countries where English is the primary language. Therefore, their English and Burmese were fluent, allowing them to provide translation and back translation services. Later, one member of the research team compared the back-translated English version with the original English scale and found three items that were different from the original instrument: I-27, “It is wrong to tell other people about this according to the rules,” I-22, “I am afraid to be criticized when others find out,” and I-45, “Some healthcare professionals are not willing to give me proper examination because I have HIV.” These three items were re-translated and back-translated. At this point, the HIVSS-M-2 was ready for pilot testing.

#### Phase 3

##### Pilot test

The HIVSS-M-2 was distributed to 10 PLWHA in Myanmar to evaluate the items’ fluency, readability, and comprehensibility. None of the participants reported confusion or incomprehension in regard to the scale items. After this process, the HIVSS-M-2 was ready for validation.

#### Phase 4

##### Psychometric test

We invited 216 PLWHA in Myanmar to complete the HIVSS-M-2; 156 PLWHA participants (72%) completed the REDCap survey. After the number of items was reduced, the reliability and validity of the HIVSS-M-3 were examined by CTT and Rasch analysis.

### Data collection

All self-reported information was collected online through the REDCap system, a web-based survey tool that is supported through the Clinical and Translational Science Institute (CTSI). Participants completed the 30-min REDCap survey, which included standardized measures to assess demographics, the HIV stigma scale, the Medical Outcomes Study–Social Support Survey (MOS-SSS; the overall Cronbach’s α for this scale in this sample was 0.96), and the Center for Epidemiological Studies Depression Scale (CES-D; the overall Cronbach’s α for this scale in this sample was 0.83). The demographic variables included participant age, gender, marital status, ethnicity, educational level, employment status, health insurance, years of living with HIV, and recent CD4 and viral load. After completing the survey, participants were reimbursed for their participation.

### Data analyses

Data analyses were conducted using IBM SPSS 23.0 (IBM, Chicago, IL, USA) and WINSTEPS 3.75.0 (Chicago, IL, USA). Missing data were replaced using the multiple imputation calculation; *p* < 0.05 was considered significant.

We first conducted item analyses and deleted an item if it met the following criteria of CTT and Rasch analysis: (a) cross-loading or factor loading < 0.4 [34], (b) infit and outfit mean squares outside the range of 0.6 to 1.4 [35], and (c) having a differential item functioning (DIF) across gender or educational level, that is, having a DIF contrast value of more than 0.43 logits and the Mantel-Haenszel analysis having statistical significance (*p* < 0.05) [35].

After item reduction, we evaluated the following reliability and validity of the HIVSS-M-3 according to the recommendations in the COSMIN checklist [30].

#### Cross-cultural validity

We used the COSMIN checklist with a 4-point scale to measure which of the descriptions on the translated scale adequately reflected the items from the original scale [30].

#### Structural validity

We combined the exploratory factor analysis (EFA) in CTT and multidimensional Rasch analysis to assess the structural validity of the scale. In the EFA, principal component analysis (PCA) and oblique rotation were used. The number of factors were extracted based on the findings of parallel analysis [34]. In multidimensional Rasch analysis, we used the rating scale model (RSM) to assess person separation reliability, person separation index, category probability curves, and person-fit statistics [36, 37]. Pearson’s fit statistics included infit and outfit mean squares, as well as difficult (location) for individual items. Furthermore, items were tested for DIF across educational levels (middle school graduation compared with each of the following: high school graduation, professional [vocational] training school graduation, some college but no degree, college graduation, and post-college graduate), and gender (male vs. female).

#### Construct validity

We estimated the convergent validity of the HIVSS-M-3 by Pearson’s correlations, with expected significant positive correlation with the CES-D and negative correlation with the MOS-SSS.

#### Internal consistency

We used Cronbachs’ α and corrected item-total correlation to assess the internal consistency of the HIVSS-M-3 [38].

#### Floor/ceiling effect

Floor effects were evaluated by examining the percentage of the respondents that achieved the lowest possible scores. Ceiling effects were evaluated by examining the percentage of respondents that reached the highest possible score.

## Results

### Sample characteristics

Of the 216 PLWHA participants, 156 (72.22%) completed the questionnaires. The mean age of participants was 28.92 years (SD = 17.32) and the average years of living with HIV was 9.57 years (SD = 5.71). The average recent CD4 count was 683.49 (SD = 475.15), and the average viral load was 615.80 (SD = 1058.55). Table 1 presents the details of the sociodemographic characteristics of the participants.

**Table 1:** Sociodemographic characteristics of the participants (*N* = 156)

Variables	*N* (%)
Gender	
Male	97 (62.20%)
Female	58 (37.70%)
Transgender	1 (0.6%)
Ethnicity	
Bamar	120 (76.9%)
Chin	2 (1.3%)
Kachin	3 (1.9%)
Kayin	7 (4.5%)
Kayah	1 (0.6%)
Mon	8 (5.1%)
Rakhine	4 (2.6%)
Shan	6 (3.9%)
Others^a^	5 (3.2%)
Marital status	
Married or steady partner	63 (40.6%)
Widowed	18 (11.6%)
Separated	6 (3.9%)
Divorced	10 (6.5%)
Single, never married	58 (37.4%)
Educational level	
Middle school graduation	16 (10.3%)
High school graduation	64 (41.0%)
Professional (vocational) training school graduation	2 (1.3%)
Some College but no degree	24 (15.4%)
College graduation	47 (30.1%)
Post college graduate	3 (1.9%)
Employment status	
No	32 (20.6%)
Part time	28 (18.1%)
Full time	96 (61.3%)
Health insurance	
Not enough	127 (81.2%)
Just enough	29 (18.8%)

^a^Palaung, Islam, Tamil

### Item retention

We found that the factor loading of item I-11 was less than 0.4, seven items (I-1, I-13,I-4, I-22, I-26,I-27, I-30) were cross-loading, and infit and outfit mean squares of three items (I-1, I-8, I-21) were outside the range of 0.6 to 1.4. In addition, two items (I-13, I-39) had a significant DIF across gender, and three items (I-1, I-27, I-31) had a significant DIF across education. According to the criteria of item retention, 12 items were deleted (see Additional file 1: Appendices A and B). Thus, the final 35-item HIVSS-M-3 was formed (see Additional file 1: Appendix C).

### Cross-cultural validity

The process of translation and the sample size (more than 150) met the requirements of “good” in the COSMIN checklist. We conducted the pilot test and formal survey to evaluate the items’ fluency, readability, and comprehensibility; all participants reported a good understanding of each item of the stigma scale.

### Structural validity

The Bartlett test of sphericity indicated that the sample was adequate for factor analysis (χ^2^ = 3672.360, df = 595, *p* < 0.001; Kaiser-Meyer-Olkin = 0.908). Based on parallel analysis, six factors were extracted with an eigenvalue of 2.00 to 6.18, together explaining 68.23% of the overall variance. Factor loadings for all items were between 0.46 and 0.86 (see Table 2). According to the original structure of Berger’s HIV stigma scale and the HIV stigma scale tested in India, the six factors were labeled (a) personalized stigma, (b) disclosure concerns, (c) negative self-image, (d) concern with public attitudes about HIV, (e) healthcare provider’s stigma, and (f) religious concerns.

**Table 2:** Factor structure of the Myanmar version of the HIV stigma scale

Items	Factors					
	1	2	3	4	5	6
Factor 1: Personalized stigma						
Cronbach’s alpha = 0.933						
I-29 People I care about stopped calling after learning	0.81					
I-18 Some people who know have grown more distant	0.79					
I-33 People have physically backed away from me	0.77					
I-38 People who know tend to ignore my good points	0.77					
I-35 Stopped socializing with some due to their reactions	0.76					
I-36 Have lost friends by telling them I have HIV	0.76					
I-32 Don’t want me around their children once they know	0.72					
I-28 People avoid touching me if they know I have HIV	0.64					
I-24 Hurt by how people reacted to learning I have HIV	0.61					
I-34 Some people act as though it’s my fault I have HIV	0.51					
Factor 2: Concerns with public attitudes about HIV						
Cronbach’s alpha = 0.939						
I-10 Most people believe a person who has HIV is dirty		0.86				
I-20 Most are uncomfortable around someone with HIV		0.85				
I-16 Most with HIV are rejected when others learn		0.82				
I-9 People with HIV are treated like outcasts		0.78				
I-40 Knowing, they look for flaws in your character		0.75				
I-14 Most people think a person with HIV is disgusting		0.72				
I-5 People with HIV lose jobs when employers learn		0.72				
Factor 3: Negative self-image						
Cronbach’s alpha = 0.914						
I-23 Having HIV in my body is disgusting to me			0.86			
I-12 Having HIV makes me feel unclean			0.83			
I-7 I feel I’m not as good as others because I have HIV			0.82			
I-15 Having HIV makes me feel I’m a bad person			0.78			
I-2 I feel guilty because I have HIV			0.76			
I-3 People’s attitudes make me feel worse about myself			0.67			
Factor 4: healthcare provider’s stigma						
Cronbach’s alpha = 0.802						
I-46 I been refused medical care or denied hospital services because I have HIV.				0.85		
I-45 A healthcare worker has not wanted to touch me because I have HIV.				0.82		
I-44 Medical provider or hospital worker have mistreated me because of my HIV.				0.75		
I-47 A hospital worker made my HIV infection publicly known by marking HIV on my medical record.				0.61		
Factor 5: Disclosure concerns						
Cronbach’s alpha = 0.836						
I-17 I am very careful whom I tell that I have HIV					0.72	
I-37 I told people close to me to keep my HIV a secret					0.65	
I-6 I work hard to keep my HIV a secret					0.64	
I-25 I worry people who know I have HIV will tell others					0.56	
I-19 I worry about people discriminating against me					0.46	
Factor 6: Religious concerns						
Cronbach’s alpha = 0.703						
I-41 I pay for karma or sins because I have HIV						0.77
I-43 People would think that I did something wrong in my last life once they know that I have HIV.						0.73
I-42 In order to end the suffering of HIV this life, I have to do good things (e.g. praying, donation).						0.67
Eigenvalue	6.18	5.52	4.85	2.69	2.65	2.00
Cumulative percentages	17.67	33.43	47.29	54.98	62.53	68.23

**Fig. 1:**
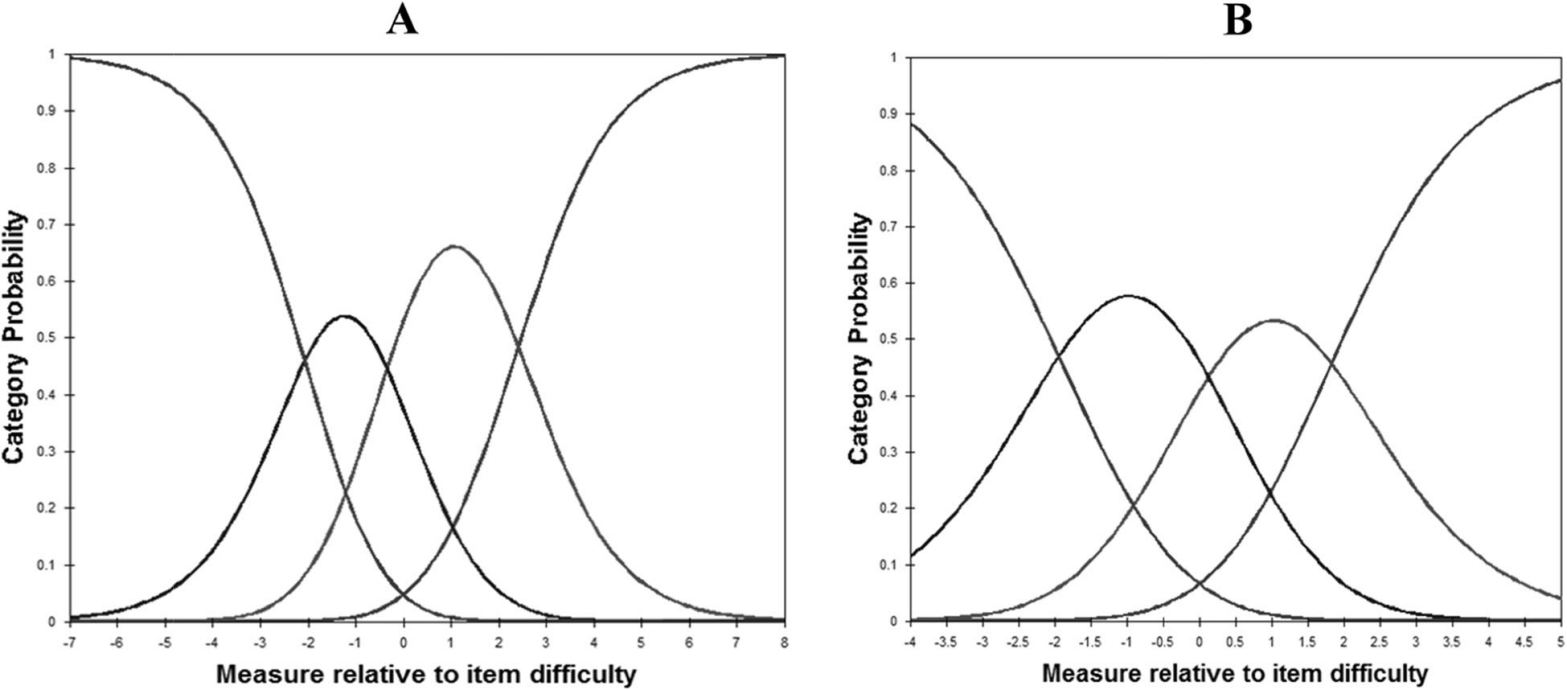
**A** Category probability curves for the item 2. **B** Category probability curves for the item 47

In the Rasch analysis, as shown in Table 3, the infit and outfit mean squares for each item ranged from 0.68 to 1.40. No evidence of disordered thresholds was found in the category probability curves, as the category calibration increased in an orderly way (see Fig. 1). We also calculated the item reliability (0.96 and 0.95), item separation index (5.15 and 4.44), person reliability (3.40 and 1.53), and person-separation index (0.92 and 0.70) in the analysis. DIF was not found when evaluated by gender and educational level.

**Table 3:** The difficult, infit, outfit MNSQ and corrected item-total correlation of 35 items

Item	Item difficult^a^	Infit MNSQ	Infit ZSTD	Outfit MNSQ	Outfit ZSTD	Corrected item-total correlation
I-2	0.53	1.12	1.2	1.24	2.0	0.57^†^
I-3	0.59	0.73	−2.8	0.73	−2.5	0.69^†^
I-5	−0.96	0.97	−0.2	0.91	−0.7	0.63^†^
I-6	−0.08	1.15	1.4	1.12	1.1	0.63^†^
I-7	0.77	0.98	−0.2	0.96	−0.3	0.62^†^
I-9	−0.61	0.74	−2.5	0.82	−1.6	0.69^†^
I-10	−0.7	0.86	−1.3	0.83	−1.5	0.65^†^
I-12	0.58	1.05	0.5	1.06	0.5	0.59^†^
I-14	−0.64	0.99	−0.1	1.01	0.1	0.65^†^
I-15	1.18	1.16	1.5	1.15	1.2	0.53^†^
I-16	−0.59	0.87	−1.2	0.83	−1.5	0.66^†^
I-17	−1.30	0.93	−0.6	0.98	−0.1	0.54^†^
I-18	0.37	0.63	−4.0	0.65	−3.6	0.74^†^
I-19	−0.25	0.81	−1.8	0.78	−2.0	0.69^†^
I-20	−0.71	0.85	−1.3	0.80	−1.8	0.66^†^
I-23	0.98	0.99	−0.1	0.98	−0.4	0.61^†^
I-24	−0.13	0.94	−0.6	0.92	−0.7	0.61^†^
I-25	−0.38	1.04	0.4	1.06	0.6	0.66^†^
I-28	−0.15	0.67	−3.4	0.66	−3.4	0.73^†^
I-29	0.40	0.75	−2.5	0.77	−2.2	0.68^†^
I-32	0.19	0.85	−1.5	0.84	−1.4	0.69^†^
I-33	0.28	0.72	−2.8	0.72	−2.8	0.70^†^
I-34	−0.09	0.85	−1.4	0.9	−0.9	0.68^†^
I-35	−0.02	0.78	−2.1	0.78	−2.1	0.68^†^
I-36	0.23	0.82	−1.8	0.81	− 1.8	0.68^†^
I-37	−0.47	1.12	1.1	1.27	1.4	0.64^†^
I-38	0.17	0.73	−2.7	0.73	−2.6	0.65^†^
I-40	−0.85	0.69	−3.1	0.66	−3.3	0.70^†^
I-41	−0.07	1.32	2.7	1.38	3.1	0.47^†^
I-42	−1.02	1.40	2.5	1.36	2.5	0.40^†^
I-43	−0.05	1.32	2.7	1.32	2.7	0.39^†^
I-44	0.10	0.84	2.1	0.87	2.3	0.33^†^
I-45	0.31	0.76	3.0	0.75	3.0	0.37^†^
I-46	1.11	1.36	3.2	1.39	2.9	0.34^†^
I-47	0.33	1.02	3.2	1.03	3.2	0.36^†^

^†^*p* < 0.05; MNSQ mean square

^a^Measured in logit; positive item logit indicates that the item requires a lower visual ability, than the mean of the items and is an easier item, whereas a negative item logit indicates that, the item requires a higher visual ability than the mean of the items and is a more difficult item

### Construct validity

The convergent validity for the HIVSS-M-3 was confirmed with positive correlation with the CES-D (r = 0.60, *p* < 0.001) and negative correlation with the MOS-SSS (r = − 0.77, p < 0.001).

### Internal consistency

The Cronbach’s alpha for the total HIVSS-M-3 was 0.95. The corrected item-total correlation ranged from 0.34 to 0.74 (*p* < 0.05).

### Floor/ceiling effect

Of the total number of participants, 1.28% (2/156) achieved the lowest possible score (35). No participant (0%) achieved the highest possible score on the scale (140). The lowest or highest possible scores were both below 15%, indicating that there were no floor or ceiling effects of the Myanmar version of the HIV stigma scale [39].

## Discussion

The present study is one of the first examinations of the constructs of HIV stigma in the Myanmar context. The Myanmar version of the HIV stigma scale was adapted and validated through a rigorous, multiphase process that followed the guidelines prescribed in the Translation and Cultural Adaptation - Principles of Good Practice [40]. Our psychometric evaluation, based on CTT and Rasch analysis, showed that the 35-item HIVSS-M-3 provides sufficient validity (cross-cultural validity, structural validity, and construct validity) and satisfactory internal consistency reliability, without a floor or ceiling effect. Therefore, the 35-item HIVSS-M-3 is a reliable and valid self-report measure for assessing stigma in PLWHA.

The factor analytic strategies used in CTT yielded a clear six-factor structure for the 35-item HIVSS-M-3. This finding confirmed that stigma differs as a construct across cultures [7, 9, 27].

The HIVSS-M-3 was adapted from the Berger HIV stigma scale [22] and the HIV stigma scale tested in India [27]. Although only 28 items of the Berger stigma scale were left in the Myanmar version, we found the same four-factor structure as previously presented by Berger et al. (2001) in an American context [22]. This finding indicates that the Berger scale can be used to measure the personalized stigma, disclosure concerns, negative self-image, and concerns regarding public attitudes among PLHWA in Myanmar. In addition, the reduced number of items further suggest the redundancy of the Berger stigma scale [23].

On the other hand, considering the culturally specific characteristics of stigma, we also adapted 7 items of the HIV stigma scale tested in India. The EFA showed that 7 items were related to the PLHWAs’ religious concerns related to HIV and to possible vicarious stigma from healthcare providers (i.e., participants relayed accounts of other people facing stigma from healthcare providers). The religious-concerns finding reflects the importance of traditional religious theology regarding accepting one’s fate among PLWHA in Myanmar. The people in Myanmar are a culturally diverse population, with 74.4% of them being Buddhist, 8.2% a Christian, 3.8% a Muslim, 1.7% Hindu, 1.5% Confucianists, and 9.5% Ethnoreligionists [41]. With the majority of Myanmars being Buddhist, the concept of karma is significant in the lives of many Myanmars. Thus, they believe in doing good things, such as praying and donating, to relieve the suffering from diseases such as HIV. In addition to the religious concerns, healthcare providers’ stigma toward PLWHA is well-represented and persists within the Myanmar healthcare system. This echoed a previous study conducted in Myanmar that found that PLWHA had been mistreated because of their HIV status [12], for example, by being placed in separate waiting areas or wards [15]. In addition, while receiving pregnancy-related services, HIV-infected women were mistreated, including being sterilized without their consent [12].

In addition to the traditional CTT methods, the structural validity of the HIVSS-M-3 was also confirmed by Rasch analysis. Our data support that the category rating scale of the HIVSS-M-3 worked well and was free of DIF by gender and educational levels. The combination of a good person-separation index (> 2) and person reliability (> 0.8) suggested that the HIVSS-M-3 has acceptable measurement precision and is sensitive to distinguishing both high and low stigma participants [37].

Similar to previous studies [9, 22, 27], the construct validity of the scale was supported, as reflected in the significant positive correlation with self-reported depressive symptomology and negative correlation with social support levels. In addition, the Cronbach’s α of more than 0.7 indicates that the HIVSS-M-3 has satisfactory internal consistency and reliability [38].

This study has several limitations. First, the sample size was relatively small and some psychometric characteristics of the HIVSS-M-3 could be assessed further, such as test-retest reliability and structural validity, and these could be checked by confirmatory factor analysis. Second, the 37.6% non-response rate and the computer literacy of participants in responding to a self-assessment survey may impact the generalizability of the findings. Third, the REDCap online platform that was used also could potentially impact the generalizability of the findings. That is, persons who could not access such a survey or who are not literate in using online surveys were not included in this study. Finally, the sensitivity of the HIVSS-M-3 was not assessed. Therefore, future longitudinal or experimental studies are warranted for checking that. A further refinement and testing of the scale using a larger representative sample would produce more stable parameter estimations and robust results.

## Conclusions

The Myanmar version of the 35-item HIV stigma scale with a six-factor structure is a sufficiently valid and reliable tool for assessing the experience and effects of stigma in PLWHA in Myanmar. Furthermore, this stigma scale could also facilitate the development of stigma-reduction interventions and evaluate the effects of such interventions.

### Relevance for clinical practice

Evidence has consistently indicated that HIV stigma is a common barrier to HIV prevention, testing, and treatment adherence [6]. Especially for low- and middle-income countries such as Myanmar, an important predictor of quality of life for PLHWA is HIV-related stigma [1]. The psychometric properties presented in this paper suggest that the 35-item HIVSS-M-3 can accurately measure the personalized stigma, disclosure concerns, negative self-image, concern with public attitudes about HIV, religious concerns, and healthcare providers’ stigma affecting PLHWA in Myanmar. This scale can also facilitate the development of stigma-reduction interventions and be used to evaluate the effects of future interventions. Future testing of the scale in more representative samples is needed to further examine the scale’s screening utility. It will also be important to determine the cut-off value for the HIVSS-M-3 and to compare the stigma faced by PLWHA in Myanmar with that faced by PLWHA globally.

## Additional file

**Additional file 1: Appendix A**. Item and factor analysis of the Berger HIV stigma scale. **Appendix B**. Item and factor analysis of the 7-item stigma scale tested in India. **Appendix C**. The HIV stigma scale in Myanmar.

## Data Availability

The original data are available on request to the corresponding author after the manuscript is published. We also plan to provide the original data to public repositories.
