# Supplementary material for: Adaptation and validation of a culturally adapted HIV stigma scale in Myanmar
Source: BMC Public Health. 2021 Sep 13;21:1663. doi: 10.1186/s12889-021-11685-w (PMC8439000; doi:10.1186/s12889-021-11685-w)
Supplement: Supplementary file 1 — Additional file 1: Appendix A. Item and factor analysis of the Berger HIV stigma scale. Appendix B. Item and factor analysis of the 7-item stigma scale tested in India. Appendix C. The HIV stigma scale in Myanmar. [file 12889_2021_11685_MOESM1_ESM.docx]

Appendix A. Item and factor analysis of the Berger HIV stigma scale

| Item | Factor loading | | | | | Infit MNSQ | Outfit MNSQ | DIF  contrast by gender^a^ | DIF  contrast by education^b,c,d,e,f^ | | | | | Item  retention |
| --- | --- | --- | --- | --- | --- | --- | --- | --- | --- | --- | --- | --- | --- | --- |
|  | Factor  1 | Factor  2 | Factor  3 | Factor  4 | Factor  5 |  |  |  |  |  |  |  |  |  |
| I29 | 0.81 |  |  |  |  | 0.85 | 0.84 | 0.33 | -0.16 | -0.21 | 0.12 | -0.41 | 0.61 | Yes |
| I18 | 0.79 |  |  |  |  | 0.65 | 0.64 | 0.00 | -0.29 | -1.43 | -0.13 | -0.47 | 0.45 | Yes |
| I33 | 0.79 |  |  |  |  | 0.78 | 0.76 | 0.19 | -0.28 | -1.39 | 0.24 | -0.31 | 0.48 | Yes |
| I38 | 0.78 |  |  |  |  | 0.79 | 0.78 | 0.08 | 0.51 | 3.19 | 0.14 | 0.66 | 0.14 | Yes |
| I36 | 0.75 |  |  |  |  | 0.90 | 0.93 | 0.47 | 0.79 | 1.33 | 0.66 | 0.54 | 1.24 | Yes |
| I35 | 0.74 |  |  |  |  | 0.83 | 0.81 | 0.06 | 0.26 | -0.32 | 0.17 | 0.00 | 0.40 | Yes |
| I24 | 0.69 |  |  |  |  | 1.02 | 1.00 | 0.50 | 0.00 | 0.32 | -0.18 | -0.03 | 0.23 | Yes |
| I32 | 0.63 |  |  |  |  | 0.89 | 1.05 | 0.35 | 0.52 | -0.89 | 0.58 | 0.37 | 0.99 | Yes |
| I28 | 0.57 |  |  |  |  | 0.69 | 0.67 | -0.35 | 0.00 | -0.59 | -0.02 | -0.05 | 0.23 | Yes |
| I26 | 0.49 |  |  |  | 0.49 | 0.97 | 0.94 | 0.10 | 0.44 | -0.89 | 0.25 | 0.11 | 0.25 | No |
| I11 | 0.39 |  |  |  |  | 0.80 | 0.78 | 0.23 | -0.13 | 0.05 | -0.61 | -0.49 | -0.04 | No |
| I10 |  | 0.86 |  |  |  | 0.97 | 0.93 | 0.09 | 0.00 | 0.92 | 0.03 | 0.22 | 0.59 | Yes |
| I20 |  | 0.86 |  |  |  | 0.94 | 0.87 | 0.09 | 0.34 | 1.19 | 0.48 | 0.37 | 1.74 | Yes |
| I16 |  | 0.81 |  |  |  | 0.92 | 0.86 | -0.04 | 0.23 | 0.19 | 0.12 | 0.44 | 0.85 | Yes |
| I40 |  | 0.79 |  |  |  | 0.81 | 0.77 | 0.10 | -0.25 | -0.50 | -0.21 | -0.11 | 0.16 | Yes |
| I9 |  | 0.77 |  |  |  | 0.82 | 0.91 | 0.03 | -0.52 | -0.50 | -0.31 | -0.48 | 0.16 | Yes |
| I5 |  | 0.76 |  |  |  | 1.08 | 1.00 | 0.34 | 0.31 | 0.92 | 0.50 | 0.43 | -0.17 | Yes |
| I14 |  | 0.71 |  |  |  | 1.11 | 1.12 | -0.25 | -0.17 | 0.88 | -0.10 | 0.24 | 0.55 | Yes |
| I23 |  |  | 0.83 |  |  | 0.99 | 0.93 | -0.22 | 0.31 | 0.73 | 0.13 | 0.35 | -1.96 | Yes |
| I12 |  |  | 0.82 |  |  | 1.03 | 1.02 | -0.55 | -0.22 | -0.10 | -0.27 | -0.22 | -0.77 | Yes |
| I7 |  |  | 0.81 |  |  | 0.92 | 0.91 | -0.10 | -0.16 | 0.04 | -0.22 | -0.39 | -2.64 | Yes |
| I2 |  |  | 0.76 |  |  | 1.10 | 1.11 | 0.55 | 0.13 | -0.46 | 0.12 | -0.10 | -0.37 | Yes |
| I15 |  |  | 0.76 |  |  | 1.15 | 1.15 | -0.18 | 0.31 | -1.36 | 0.61 | 0.31 | -1.73 | Yes |
| I3 |  |  | 0.63 |  |  | 0.75 | 0.75 | -0.24 | 0.25 | 1.54 | 0.65 | 0.63 | 3.30 | Yes |
| I6 |  |  |  | 0.67 |  | 1.08 | 1.04 | -0.62 | -0.39 | 0.19 | -0.22 | -0.04 | -0.63 | Yes |
| I37 |  |  |  | 0.62 |  | 1.14 | 1.21 | -0.34^†^ | 0.22 | -0.87 | -0.38 | -0.10 | 0.71 | Yes |
| I4 |  | 0.41 |  | 0.62 |  | 0.92 | 0.87 | -0.10 | 0.13 | 0.19 | -0.23 | 0.24 | -0.63 | No |
| I1 |  |  | 0.45 | 0.62 |  | 1.39 | 1.57 | -0.30^†^ | -0.36^†^ | -0.32 | -0.20 | -0.27 | -0.40 | No |
| I25 |  |  |  | 0.60 |  | 1.04 | 1.13 | -0.03 | 0.25 | 0.40 | 0.42 | 0.46 | -2.05 | Yes |
| I17 |  |  |  | 0.60 |  | 0.95 | 0.89 | -0.12 | -0.16 | 1.45 | -0.22 | 0.14 | -0.13 | Yes |
| I22 |  | 0.50 |  | 0.57 |  | 0.71 | 0.68 | -0.30 | 0.23 | 1.19 | -0.43 | 0.24 | 0.85 | No |
| I21 |  |  |  |  | 0.87 | 2.30 | 3.90 | 0.13 | 1.01 | 1.49 | 0.49 | 0.72 | -0.96 | No |
| I8 |  |  |  |  | 0.77 | 2.22 | 3.17 | 0.29 | 0.25 | 0.02 | -0.22 | -0.08 | -1.51 | No |
| I34 |  |  |  |  | 0.67 | 0.87 | 0.85 | 0.08 | -0.56 | -2.05 | -0.41 | -0.51 | -0.17 | Yes |
| I31 |  |  |  |  | 0.66 | 0.87 | 0.82 | 0.21 | 0.32 | -0.03 | 0.43 | 0.47^†^ | 0.68 | No |
| I13 | 0.46 |  |  |  | 0.50 | 1.04 | 1.05 | 0.59^†^ | -0.77 | -1.52 | -0.22 | -0.57 | -0.37 | No |
| I19 |  |  |  |  | 0.46 | 0.82 | 0.78 | -0.31 | -0.58 | -0.79 | -0.80 | -0.20 | 0.03 | Yes |
| I27 | 0.42 |  |  |  | 0.43 | 0.65 | 0.68 | 0.00 | 0.61^†^ | 0.11 | 0.32 | -0.14 | 0.03 | No |
| I30 | 0.41 |  |  |  | 0.53 | 1.20 | 1.24 | -0.23 | -0.03 | -1.14 | 0.24 | -0.29 | 0.01 | No |
| I39 |  |  |  |  | 0.43 | 0.90 | 0.90 | -0.63^†^ | -0.54 | -0.68 | -0.84 | -0.11 | 0.03 | No |

^†^*p*<0.05

MNSQ: mean squares; DIF: differential item functioning.

^a^male compared with female.

The DIF contrast by education in the following order:

^b^middle school graduation compared with high school graduation.

^c^middle school graduation compared with professional (vocational) training school graduation.

^d^middle school graduation compared with some college but no degree.

^e^middle school graduation compared with college graduation.

^f^middle school graduation compared with post college graduate.

Appendix B. Item and factor analysis of the 7-item stigma scale tested in India

| Item | Factor loading | | Infit MNSQ | Outfit MNSQ | DIF contrast by gender^a^ | DIF contrast by education^b,c,d,e,f^ | | | | | Item  retention |
| --- | --- | --- | --- | --- | --- | --- | --- | --- | --- | --- | --- |
|  | Factor 1 | Factor 2 |  |  |  |  |  |  |  |  |  |
| I45 | 0.87 |  | 0.76 | 0.75 | -0.94 | 0.30 | 1.33 | 0.31 | 0.19 | 0.78 | Yes |
| I46 | 0.83 |  | 0.86 | 0.83 | -0.77 | -0.51 | -0.96 | -0.39 | -0.56 | -0.89 | Yes |
| I44 | 0.82 |  | 0.84 | 0.87 | -0.73 | 0.20 | -0.17 | 0.04 | -0.19 | 0.35 | Yes |
| I47 | 0.61 |  | 1.02 | 1.03 | 0.50 | 0.50 | 1.63 | 0.74 | 0.64 | 0.33 | Yes |
| I41 |  | 0.82 | 1.10 | 1.13 | 0.44 | -0.23 | -0.73 | -0.08 | 0.07 | -.022 | Yes |
| I43 |  | 0.78 | 1.03 | 1.03 | 1.14 | 0.29 | -1.33 | 0.18 | 0.52 | 0.21 | Yes |
| I42 |  | 0.76 | 1.40 | 1.36 | 0.38 | -0.62 | 0.34 | -0.85 | -0.74 | -0.65 | Yes |

MNSQ: mean squares; DIF: differential item functioning.

^a^men compared with female.

The DIF contrast by education in the following order:

^b^middle school graduation compared with high school graduation.

^c^middle school graduation compared with professional (vocational) training school graduation.

^d^middle school graduation compared with some college but no degree.

^e^middle school graduation compared with college graduation.

^f^middle school graduation compared with post college graduate.

Appendix C .The HIV stigma scale in Myanmar

| Item | Strongly disagree | Disagree | Agree | Strongly agree |
| --- | --- | --- | --- | --- |
| 2. I feel guilty because I have HIV | 1 | 2 | 3 | 4 |
| 3. People's attitudes about HIV make me feel worse about myself | 1 | 2 | 3 | 4 |
| 5. People with HIV lose their jobs when their employers find out | 1 | 2 | 3 | 4 |
| 6. I work hard to keep my HIV a secret | 1 | 2 | 3 | 4 |
| 7. I feel I am not as good a person as others because I have HIV | 1 | 2 | 3 | 4 |
| 9. People with HIV are treated like outcasts | 1 | 2 | 3 | 4 |
| 10. Most people believe that a person who has HIV is dirty | 1 | 2 | 3 | 4 |
| 12. Having HIV makes me feel unclean | 1 | 2 | 3 | 4 |
| 14. Most people think that a person with HIV is disgusting | 1 | 2 | 3 | 4 |
| 15. Having HIV makes me feel that I'm a bad person | 1 | 2 | 3 | 4 |
| 16. Most people with HIV are rejected when others find out | 1 | 2 | 3 | 4 |
| 17. I am very careful who I tell that I have HIV | 1 | 2 | 3 | 4 |
| 18. Some people who know I have HIV have grown more distant | 1 | 2 | 3 | 4 |
| 19. Since learning I have HIV, I worry about people discriminating against me | 1 | 2 | 3 | 4 |
| 20. Most people are uncomfortable around someone with HIV | 1 | 2 | 3 | 4 |
| 23. Having HIV in my body is disgusting to me | 1 | 2 | 3 | 4 |
| 24. I have been hurt by how people reacted to learning I have HIV. | 1 | 2 | 3 | 4 |
| 25. I worry that people who know I have HIV will tell others | 1 | 2 | 3 | 4 |
| 28. Some people avoid touching me once they know I have HIV | 1 | 2 | 3 | 4 |
| 29. People I care about stopped calling after learning I have HIV | 1 | 2 | 3 | 4 |
| 32. People don't want me around their children once they know I have HIV | 1 | 2 | 3 | 4 |
| 33. People have physically backed away from me when they learn I have HIV | 1 | 2 | 3 | 4 |
| 34. Some people act as though it's my fault I have HIV | 1 | 2 | 3 | 4 |
| 35. Some people avoid touching me once they know I have HIV | 1 | 2 | 3 | 4 |
| 36. I have lost friends by telling them I have HIV. | 1 | 2 | 3 | 4 |
| 37. I have told people close to me to keep the fact that have HIV a secret | 1 | 2 | 3 | 4 |
| 38. People who know I have HIV tend to ignore my good points | 1 | 2 | 3 | 4 |
| 40. When people learn you have HIV, they look for flaws in your character | 1 | 2 | 3 | 4 |
| 41. I pay for karma or sins because I have HIV. | 1 | 2 | 3 | 4 |
| 42. People would think that I did something wrong in my last life once they know that I have HIV. | 1 | 2 | 3 | 4 |
| 43. A healthcare worker has not wanted to touch me because I have HIV. | 1 | 2 | 3 | 4 |
| 44. Medical provider or hospital worker have mistreated me because of my HIV. | 1 | 2 | 3 | 4 |
| 45. A healthcare worker has not wanted to touch me because I have HIV. | 1 | 2 | 3 | 4 |
| 46. I been refused medical care or denied hospital services because I have HIV. | 1 | 2 | 3 | 4 |
| 47. A hospital worker made my HIV infection publicly known by marking HIV on my medical record. | 1 | 2 | 3 | 4 |
